# *Staphylococcus aureus* α-Toxin: Nearly a Century of Intrigue

**DOI:** 10.3390/toxins5061140

**Published:** 2013-06-13

**Authors:** Bryan J. Berube, Juliane Bubeck Wardenburg

**Affiliations:** 1 Department of Microbiology, The University of Chicago, 920 E. 58th Street Chicago, IL 60637, USA; E-Mail: bberube@bsd.uchicago.edu; 2 Department of Pediatrics, The University of Chicago, 5721 S. Maryland Ave. Chicago, IL 60637, USA

**Keywords:** α-toxin, *Staphylococcus aureus*, pore-forming toxins, ADAM10, cellular responses, *S. aureus* vaccine and therapeutic

## Abstract

*Staphylococcus aureus* secretes a number of host-injurious toxins, among the most prominent of which is the small β-barrel pore-forming toxin α-hemolysin. Initially named based on its properties as a red blood cell lytic toxin, early studies suggested a far greater complexity of α-hemolysin action as nucleated cells also exhibited distinct responses to intoxication. The hemolysin, most aptly referred to as α-toxin based on its broad range of cellular specificity, has long been recognized as an important cause of injury in the context of both skin necrosis and lethal infection. The recent identification of ADAM10 as a cellular receptor for α-toxin has provided keen insight on the biology of toxin action during disease pathogenesis, demonstrating the molecular mechanisms by which the toxin causes tissue barrier disruption at host interfaces lined by epithelial or endothelial cells. This review highlights both the historical studies that laid the groundwork for nearly a century of research on α-toxin and key findings on the structural and functional biology of the toxin, in addition to discussing emerging observations that have significantly expanded our understanding of this toxin in *S. aureus* disease. The identification of ADAM10 as a proteinaceous receptor for the toxin not only provides a greater appreciation of truths uncovered by many historic studies, but now affords the opportunity to more extensively probe and understand the role of α-toxin in modulation of the complex interaction of *S. aureus* with its human host.

## 1. Introduction

*Staphylococcus aureus* α-hemolysin (α-toxin, Hla) is the prototype for the class of small β-barrel pore-forming cytotoxins (PFTs) [1,2,3,4]. *S. aureus* α-toxin is secreted as a water soluble monomer, capable of binding and oligomerization into a heptameric structure on the host cell membrane [5,6]. This molecular transformation on susceptible host cells culminates in the extension of a membrane-perforating 1–3 nm β-hairpin lined amphipathic pore through the eukaryotic lipid bilayer, allowing for the flow of Ca^2+^ and K^+^, ATP, and low molecular weight molecules with a cutoff between 1 and 4 kDa through the barrel of the pore [1]. While pore formation and cellular lysis are a prominent consequence of α-toxin action, a number of studies in recent years have defined cellular responses to sublytic intoxication, notably the alteration of cell signaling pathways that govern cell proliferation, inflammatory responses, cytokine secretion, and cell-cell interactions (extensively reviewed in [1,7]; see also [8,9,10,11,12,13]). 

For many years the relevance of α-toxin-mediated injury to human disease was the subject of debate as multiple investigations focused on the exquisite susceptibility of rabbit erythrocytes to lysis, in contrast to a *relative* insensitivity of human red cells [14,15,16,17,18]. However, in 1964, Siegel and Cohen demonstrated that α-toxin causes the aggregation of human platelets at sublytic concentrations [19]. Since then, α-toxin has been shown to intoxicate a wide range of human cell types, including epithelial cells, endothelial cells, and an array of other hematopoietic-lineage cells including T cells, monocytes, macrophages, and neutrophils [1,7,13,18,20,21,22,23,24]. Further, multiple studies have investigated the human and small animal host response to the toxin, both shedding light on how this toxin causes injury and defining salient features of the cellular and organismal response to the toxin [9,11,13,20,25,26,27,28,29,30,31,32,33].

*S. aureus* α-toxin has been the subject of a number of exceptional reviews that provide a detailed record of the many studies that have contributed to our current knowledge of the toxin; we recommend these to the reader [1,7,34,35,36]. In this review, we will highlight key observations on α-toxin that illustrate the defining features of toxin biology and its role in disease pathogenesis. Given the common use of pore-forming toxins by bacterial pathogens, it is anticipated that the ever-increasing knowledge of *S. aureus* α-toxin will likely provide greater insight on the biologic function of this family of toxins. While many early investigations on α-toxin lack the sophisticated experimental techniques currently available, these observations can now be viewed in light of our existing knowledge to have provided extraordinary fundamental insights on *S. aureus* disease and α-toxin-mediated injury. These seminal discoveries have been validated over decades of research, now expanded in scope through newer observations that provide molecular detail of toxin action and more clearly define the contribution of α-toxin to *S. aureus* disease pathogenesis. Our wealth of insight on this toxin highlights interesting new areas for investigation and defines the potential to target α-toxin through preventive and therapeutic strategies to combat human *S. aureus* disease, both of which will be explored in this review.

## 2. Historic Studies

Investigation on the toxic activity of staphylococcal supernatants began in the late 1800s. These initial studies attributed lethality in guinea pigs and rabbits, dermonecrosis, inflammation of the conjuctival epithelium, and hemolysis to toxigenic substances secreted by *S. aureus* [37,38,39,40,41,42,43]. However, the precipitating event that sparked a rigorous examination of *S. aureus* and its toxins came in the late 1920s following a tragedy in Bundaberg, Australia [14,35,44]. Twenty-one children in that town were immunized with a diphtheria toxin-antitoxin preparation. Within hours, 16 children experienced vomiting, high fever, unconsciousness, and convulsions. Within two days, 12 of the children had died, while all of the surviving children developed abscesses at the site of the immunization. F. McFarlane Burnet, then in the early days of his career, was commissioned by the Commonwealth of Australia to further investigate the cause of this tragedy. The Royal Commission noted in their investigation that, “massive production of toxic substances must have taken place if staphylococci were the responsible agents” [44]. Burnet discovered that culture supernatants from the vaccine-contaminating *S. aureus* strain caused hemolysis and lethal injury upon injection into rabbits [14,15,44,45]. Further, he provided a cohesive analysis of other *S. aureus* isolates that had been investigated at that time for their toxic properties, concluding that a single, heat-labile antigenic substance secreted by this pathogen was responsible for multiple biologic effects including hemolysis *in vitro*, dermonecrosis upon intradermal inoculation *in vivo*, and acute death upon injection into a rabbit. Burnet and his contemporaries also made the key observation that active immunization with formalin-treated supernatant preparations or passive immunization with antitoxin containing serum derived from immune rabbits afforded protection against disease in naïve rabbits and neutralized hemolytic and necrotic activity [14,15].

In the years immediately following Burnet’s studies, Glenny and Stevens described two immunologically distinct toxins secreted by *S. aureus* that displayed species-specific hemolytic activity. They designated the rabbit-specific toxin as α-toxin [46]. Over the next few decades, crude preparations of α-toxin from staphylococcal supernatants led to several significant observations. In particular, rabbit erythrocytes were shown to be exquisitely sensitive to hemolysis by α-toxin [47,48,49]. This sensitivity was paralleled by comparative toxicity studies in a number of small animals, which demonstrated that rabbits succumb to the lethal effects of the toxin at an LD_50_ of 2 μg/kg body weight, the lowest of any species tested [34]. 

In the 1960s, isolation of purified ãtoxin from culture supernatants allowed for a wide range of structural, biochemical, and cellular biological experiments to be performed [50,51], tremendously advancing knowledge of this toxin and more broadly, the pore-forming family of toxins. Early experiments with purified α-toxin indicated the toxin might function by disrupting host cell membranes, initially noted by Bernheimer and Schwartz who stated, “In view of the rapidity with which it brings about cell damage and in view of its remarkable lytic action on red blood cells, as distinct from diphtheria, tetanus, and botulinum toxin which have neither of these properties, perhaps the best hypothesis is that it alters or disrupts cell membranes” [51]. Consistent with this hypothesis, low molecular weight markers of <1–2 nm in size leaked out of toxin-treated cells [52,53,54,55], oligomeric structures could be isolated from red cell membrane preparations [56], and membrane lesions could be visualized in the plasma membrane of toxin-treated cells [57]. While these effects were not specific to rabbit erythrocytes, the molecular mechanism by which α-toxin exhibited selective cytotoxicity across a wide array of cells remained a focus of investigation for many years.

## 3. Properties of α-Toxin

### 3.1. Toxin Structure and Regulation of Production

The gene coding for α-toxin was discovered in the early 1980s utilizing a recombinant phage-based strategy that transferred the ability to lyse red blood cells to *E. coli* [58]. Further studies narrowed the region responsible for toxin activity to a 1620 base pair genomic DNA fragment [59]; the full DNA coding and protein sequence being identified shortly after in 1984 [60]. Present in a single copy on the staphylococcal chromosome, the *hla* locus is rather invariant across sequenced *S. aureus* strains, with almost complete conservation of primary amino acid sequence. The *hla* locus encodes a 319 amino acid protein containing a 26 amino acid leader peptide predicted to be α-helical in structure [60]. The polypeptide is processed to yield a mature extracellular protein of 293 amino acids weighing approximately 33 kDa [61]. Circular dichroism studies revealed the mature toxin is composed almost entirely of β-strands with little to no α-helical structure [62]. 

**Figure 1:**
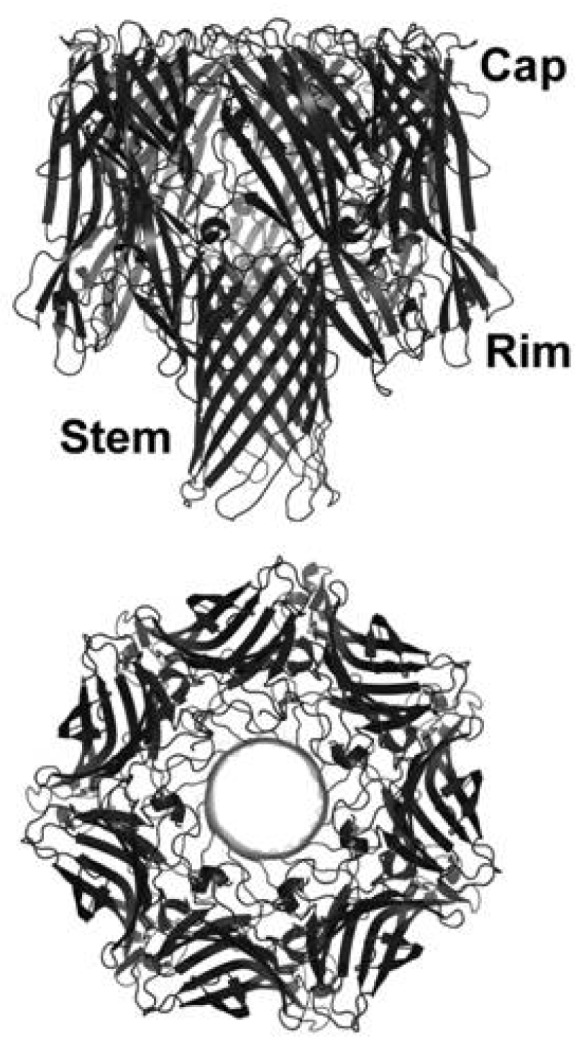
Structure of α-toxin. Crystal structure of α-toxin derived from the RCSB Protein Data Bank (PDB, 7AHL) and prepared using PYMOL, noting the regions of the toxin that demarcate the entry of the pore (Cap), the membrane-interfacing region (Rim), and the membrane perforating stem.

Early evidence suggested that α-toxin monomers aggregated into an oligomeric structure on the host cell surface. Electron micrographic images of Hla-treated cells or artificial liposomes led to the discovery of ring-like structures 10 nm in diameter with 6–7 subunits and a central pore of approximately 2–3 nm [56,57,63,64,65,66,67]. Initial biochemical studies, including purification of membrane-bound oligomers, led to the determination that α-toxin formed stable hexamers [56,68,69,70]. Gouaux and colleagues clarified these observations, employing X-ray diffraction analysis to propose a heptameric toxin structure [6]. This heptameric structure was confirmed in 1996 when Song and colleagues solved the crystal structure of the fully assembled toxin pore (Figure 1, [5]). The holotoxin is described to encompass three broad domains: (1) the cap domain on the extracellular face of the toxin, exposed to the aqueous environment, defining the entry of the pore; (2) the rim domain that is juxtaposed to the outer leaflet of the host plasma membrane; and (3) the stem domain that forms the membrane-perforating β-barrel pore [5]. 

Expression of the α-toxin monomer is controlled by several global regulatory systems [71]. The accessory gene regulator (*agr*) locus, codes for a quorum-sensing system that provides the primary control of Hla production via a regulatory RNA molecule, RNAIII [72,73]. Activated during late-log and stationary phases of growth, the *agr* system enables the production of the secreted autoinducer peptide (AIP). AIP binding to its cell surface, AgrC, activates its response regulator, AgrA [74,75]. AgrA binds to the P3 promoter of the *agr* locus and activates the production of the RNAIII molecule [76], culminating in the increased expression and secretion of *hla* with only 1% of total α-toxin remaining cell-associated [73,77]. While this system provides the primary mechanism of regulation of *hla*, expression levels can also be modulated by both the Sae and Sar regulatory systems [78,79,80,81]. Despite the challenges associated with determining the contribution of these regulatory circuits *in vivo*, it is clear this complex interplay between these global regulators allows for the tight control of *hla* expression and likely facilitates a rapid yet specific response to changing environmental conditions.

### 3.2. Host Cell Binding

The molecular mechanism by which α-toxin binds to the surface of host cell membranes had been a longstanding subject of debate in the field [17,18,82], as experimental evidence provided by multiple investigators either supported the ability of the toxin to bind to membrane lipids or to interact with host cells in a specific fashion consistent with proteinaceous receptor binding. Lending support to the former mechanism, (1) α-toxin binds to artificial lipid membranes, and can perforate lipid vesicles leading to the release of intravesicular contents [52,66,83,84,85,86,87,88,89]; (2) the “rim-stem crevice” of the toxin directly interacts with membrane lipids [90]; (3) cholesterol depletion abrogates binding of α-toxin to host cell membranes [82]; and (4) the addition of exogenous phosphocholine antagonizes toxin binding [82]. Further, multiple bacterial pore-forming cytotoxins utilize membrane lipids as their cellular receptors establishing a precedent for this mode of interaction [91]. These results, however, failed to explain the exquisite cell type and species specificity for α-toxin binding and intoxication, highlighted by the drastic difference in susceptibility to Hla-mediated lysis between rabbit erythrocytes (with lysis occurring in the low nanomolar range) and human erythrocytes (with lysis occurring in the high nanomolar to low micromolar range) [18]. By performing detailed analysis of host cell binding using radioiodinated toxin, Cassidy and Harshman determined that α-toxin binding to rabbit erythrocytes was saturable with a dissociation constant (*K*_d_) of 6 × 10^−9^ at 20 °C [17]. They estimated approximately 5000 discrete toxin-binding sites per red cell, and therefore, argued for the existence of a high-affinity receptor for α-toxin. This notion was extended by the findings of Hildebrand and colleagues indicating there are ~1500–2000 high-affinity “receptors” on sensitive cells, resulting in half-maximal toxin binding at 2 nM, while non-susceptible cells were subject to adsorptive binding of Hla with lysis observed only at high toxin concentrations [18]. 

For over a decade, the elusive nature of the proteinaceous toxin receptor coupled with the demonstrated ability of α-toxin pores to form in a purified lipid membrane cast considerable doubt on the necessity or relevance of a protein dock on susceptible cells. Valeva and colleagues aimed to unite these seemingly disparate findings by hypothesizing that clustered phosphocholine headgroups serve as the high-affinity receptor for α-toxin [82]. Their investigations revealed that treatment of cells with sphingomyelinase considerably diminished toxin binding, as did depletion of cellular cholesterol. Interesting recent observations on the role of *S. aureus*-elaborated membrane vesicles (MV, akin to outer membrane vesicles described in Gram negative bacteria) indicate that α-toxin can be delivered to the eukaryotic cell packaged in MVs, also requiring cholesterol in the target cell membrane to facilitate MV fusion and α-toxin action [92,93]. 

While these findings failed to explain the observed species specificity of toxin action on erythrocytes, a lipid-receptor hypothesis comprised the prevailing thought in the field until a few years ago when ADAM10 was defined as a candidate proteinaceous receptor for α-toxin [94]. Taking advantage of the differential susceptibility of rabbit and human erythrocytes to lysis, A Disintegrin And Metalloprotease 10 (ADAM10) was determined to be a proteinaceous receptor for α-toxin, supported by the following: (1) ADAM10 is precipitated by Hla from the membrane of host cells; (2) ADAM10 is required for toxin binding and oligomerization; (3) the requirement for ADAM10 in Hla-mediated cytotoxicity is most apparent at low toxin concentrations wherein the need for a high-affinity cellular receptor was predicted to be most relevant [17]; and (4) the species specificity exhibited by α-toxin was demonstrated to correlate with ADAM10 expression on rabbit erythrocytes, in contrast to its absence on the surface of the human red cell [94]. The observed interactions of α-toxin with both membrane lipids and a proteinaceous receptor indicate the probable cooperativity of these interactions in modulation of toxin binding, assembly and cytotoxicity. 

### 3.3. Oligomerization and Pore Formation

The transforming structural events that result in perforation of the host lipid bilayer by α-toxin has been the study of intensive investigation, taking advantage of biochemical and structural analytic tools to define the movements of discrete protein segments into the membrane to generate the pore. α-Toxin exhibits a well-defined pre-pore state, representing the fully assembled oligomeric structure on the host cell membrane. As the transition from monomeric toxin through the pre-pore state to the open pore has been the subject of extensive review within the structural biology field, we refer the reader to several excellent reviews [36,95].

Several notable observations have arisen subsequent to these reviews, and have solidified a path that allows for structure-function insights to be utilized in the development of disease modifying strategies. The α-toxin polypeptide does not contain any cysteine residues, a fact that has been capitalized on by a number of studies in which the introduction of cysteine residues has permitted both discrete site labeling for assessment of toxin structure and the generation of “locked” mutants that are unable to form membrane-inserted pores [96,97,98]. Valeva and colleagues determined that the *N*-terminal segment of α-toxin undergoes a conformational shift to “latch” onto the neighboring protomer, stabilizing the heptameric pore structure [97]. The importance of the *N*-terminus was further demonstrated as the His_35_ residue precipitates the insertion of the stem domain into the membrane by moving into a hydrophobic environment at the pre-pore to pore transition [96]. Consequently, substitution mutants at this residue are unable to form a stable oligomer, and are thus incapable of assembly into a lytic pore in spite of their preservation of cellular binding ability [96,99,100,101,102]. Further underscoring the importance of the *N*-terminus in regulation of toxin assembly, a series of *N*-terminal truncation mutants encompassing up to the first 18 residues was consistent with a role for this portion of the protein in preventing the premature oligomerization of the monomeric toxin in solution [103]. 

Several lines of evidence illustrate the magnitude of the conformational change that occurs between the monomer-pore transition. Kawate and Gouaux engineered a tether between the pre-stem domain and the cap of the toxin by the incorporation of cysteine residues at amino acids 104 and 154 [98]. This molecule is “arrested” as a non-injurious heptameric pre-pore under oxiding conditions, resolving to a fully lytic pore under reducing conditions. Several years later, the structure of the water-soluble α-toxin monomer was reported [104], providing further insight on the mechanism of conversion of the pre-pore to the assembled pore. While the small angle X-ray scattering technique utilized to obtain this structure suffers from low resolution, this technique was combined with molecular modeling to reveal that many features of the monomeric structure are conserved in the heptamer. The transitions from soluble monomeric toxin to the fully assembled pore are therefore discernable as distinct conformational entities, with the pre-pore to pore transition representing an irreversible molecular event. Several significant movements or regions of flexibility are noted in the transition process from monomeric toxin to the open pore, consistent with prior biochemical observations: (1) the rather dramatic down-folding of the central glycine-rich β-hairpin stem from each subunit (Lys_100_–Tyr_148_) away from the monomer to perforate the membrane; (2) extension of the *N*-terminal segment (Ala_1_–Val_20_) toward the neighboring protomer in the assembled heptamer, stabilizing the structure; and (3) flexibility of the phosphocholine binding region at the rim-stem interface [104]. These findings were supported by the recent demonstration of the structure of the α-toxin monomer complexed to a neutralizing monoclonal antibody [105]. The wealth of insight derived from structure-function studies of pore formation has focused considerable interest on His_35_ and other non-toxinogenic mutants as candidates for vaccine development, as well as monoclonal antibody strategies that impede toxin action through specific effects on conformational changes that occur in the molecule or through blocade of toxin binding [105,106,107,108]. 

## 4. Contribution of α-Toxin to *S. aureus* Disease

The Bundaberg accident and related investigations were the first of multiple studies to suggest that α-toxin may play an important role in the pathogenesis of human disease, now primarily supported through two lines of evidence. First, carriers of *S. aureus* or individuals suffering from *S. aureus* disease develop serum antibody responses to the toxin consistent with toxin expression [26,27,109]. While these investigations do not provide a direct correlation between serum antibody titer and disease outcome, two recent studies begin to address this issue. Adhikari and colleagues examined a population of 100 adults at risk for *S. aureus* sepsis, revealing that the risk of sepsis was reduced in individuals with higher serum antibody titers to Hla and a collection of 4 other *S. aureus* toxins [109]. Fritz and colleagues examined serum anti-Hla responses in 235 children categorized in four cohorts—*S. aureus* colonized without evidence or history of infection, primary skin/soft tissue infection, recurrent skin/soft tissue infection, and invasive *S. aureus* disease. Children with invasive disease developed higher anti-Hla antibody titers, suggestive of toxin exposure. Of considerable interest, enrollees received one-year follow-up to examine the relationship between antibody titers and protection against *S. aureus* skin infection. Throughout the follow-up period, a statistically significant increase in anti-Hla titer correlated with protective immunity against recurrent infection [27]. 

Bacterial genetic and protein profiling analysis provides a second line of evidence implicating α-toxin in the pathogenesis of human disease. The so-called “Phage-type 80/81” epidemic of the 1950s and 1960s was notable for rampant and severe *S. aureus* disease in the population, afflicting individuals with an array of clinical manifestations of disease including skin/soft tissue infection, pneumonia, and sepsis/bacteremia [110,111,112]. Analysis of the *hla* and *agr* loci in these strains revealed the capability for α-toxin expression, confirmed by a highly virulent phenotype of these isolates in animal studies of Hla-mediated disease [113]. In contrast, these investigators demonstrated that current hospital infection isolates (lineage EMRSA-16 and related clones that cluster in the same clonal complex) harbor point mutations in both loci, preventing α-toxin production. These strains exhibit a corresponding decrease in virulence observed in animal models. Consistent with these findings, current epidemic USA300 isolates of *S. aureus* that cause a significant disease burden in healthy human hosts display both increased Hla expression and virulence in experimental models, dependent on the Agr and Sae regulatory systems that govern toxin expression [114,115,116]. In addition, α-toxin expression was associated with non-resolution of bacterial peritonitis in individuals receiving peritoneal dialysis [117]. Together, these studies are most consistent with the conclusion that α-toxin expression may be required for the pathogenesis of invasive disease in healthy individuals, while of lesser relevance in individuals that are already predisposed to invasive bacterial infection on account of underlying illness, hospitalization, or tissue barrier compromise in the setting of indwelling medical devices [113].

Advances in *S. aureus* genetic manipulation have allowed for perturbation of α-toxin expression and a rigorous analysis of its role in the pathogenesis of disease in experimental animals. The use of toxin-deficient strains has highlighted the diversity of organs and tissue systems in which α-toxin plays a significant role, as Hla-deficient mutants display reduced virulence in animal models of pneumonia [20,28], dermonecrotic skin infection [30,118], sepsis [13,119], peritonitis [118,120,121], and infection of the cornea [33], central nervous system [32], endocardium [122], and the mammary gland [123,124].

**Figure 2:**
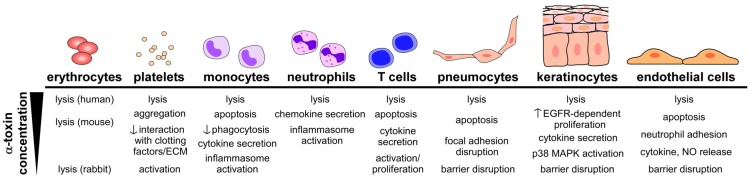
Cellular responses to intoxication by Hla. Multiple cell types are targeted by α-toxin, each displaying unique effects that are dependent on the relative concentration of toxin to which the cell is exposed.

While early studies alluded to the triad of lethal disease, hemolysis, and dermonecrosis as the chief manifestations of α-toxin-induced host injury [14], clinical and disease modeling data highlight a considerable complexity of the role of α-toxin in pathogenesis consistent with the ability of the toxin to cause injury and elicit cellular responses in a wide array of cell types (Figure 2). The discovery of ADAM10 as a cellular receptor for α-toxin has allowed for a more thorough examination of the molecular mechanisms by which α-toxin contributes to disease at the epithelial and endothelial tissue barriers [11,12,13]. These findings, along with substantial recent advances in our understanding of toxin-mediated regulation of host immunity [9,29,31,125,126,127,128], will be the subject of this section.

### 4.1. Toxin-Induced Tissue Injury

ADAM10, a cellular receptor for α-toxin, is a zinc-dependent metalloprotease expressed as a type I transmembrane protein on the surface of a wide array of host cells [129,130]. The extracellular domain of ADAM10 is comprised of an *N*-terminal enzymatic domain followed by the so-called “disintegrin” and cysteine-rich domains, both of which may facilitate protein-protein interactions at the cell surface. The short cytoplasmic tail of ADAM10 encompasses a proline-rich sequence and a consensus binding site for calmodulin [131]. Functioning as a cellular “sheddase”, ADAM10 is responsible for the cleavage of the ectodomains of a large number of host proteins including members of the cadherin family, epithelial growth factor, betacellulin, syndecans, chemokines, amyloid precursor protein (APP), platelet glycoprotein VI, notch, and ephrin, with substrate specificity varying by cell type [132,133,134,135,136,137,138,139,140]. Cleavage by ADAM10 leads to ectodomain release from the cell surface and the retention of a membrane-bound fragment, which is subject to further proteolytic processing. ADAM10-mediated cleavage yields discrete biologic outcomes, as many extracellular and intracellular cleavage products are active signaling moieties that facilitate both physiologic and pathophysiologic processes.

The requirement for ADAM10 as a cellular receptor for α-toxin in *S. aureus* pathogenesis was recently demonstrated utilizing conditional knockout approaches in the alveolar epithelium and the mature epidermis [11,12]. Germline deletion of ADAM10 results in embryonic lethality at E9.5 [141], and is associated with cell-specific abnormalities upon conditional knockout the developing epidermis, neuronal progenitor cells, the endothelium, and several hematopoietic lineage cells [142,143,144,145,146,147]. The loss of ADAM10 expression in the lung led to a marked improvement in the outcome of *S. aureus* pneumonia [11], minimizing lethal disease as compared to control mice. Similarly, ADAM10 knockout in the skin was associated with a reduction in the size of *S. aureus* skin lesions and abrogation of the severe dermonecrotic tissue injury that is a hallmark of the action of α-toxin [12]. 

A number of studies indicate that α-toxin induces signaling events in the target cell (Figure 2). The small pore formed by the toxin permits the rapid release of ATP, K^+^ ions (or ^86^Rb^+^, a K^+^ analogue) [17,128,148], however, restricts the movement of macromolecules across the cell membrane. One of the early and perhaps most important, cellular events following toxin pore formation is the influx of extracellular calcium into the cell. As a central trigger of cell signaling pathways, increased intracellular calcium stimulates hydrolysis of membrane phospholipids and metabolism of arachadonic acid to leukotrienes, prostanoids, and thromboxane A2 [21,149,150]. Toxin treatment also leads to the generation of nitric oxide in endothelial and epithelial cells, activation of protein kinase C, and induction of NF-κB nuclear translocation [21,150,151]. Together, these events signify the pro-inflammatory stimulus evoked by intoxication, also evident by cellular production of IL-1β, IL-6, and IL-8 [148,150]. These inflammatory stimuli, as well as associated cell death via pyroptosis, can exert a marked impact on the local tissue microenvironment, stimulating immune cell recruitment, increasing reactivity of the vasculature, promoting tissue edema, and modulating host immunity (further discussed in Section 4.2 below) [9,152,153,154]. It is attractive to hypothesize that one or more domains of ADAM10, particularly the transmembrane domain or cytoplasmic tail of the molecule, may contribute to toxin-induced intracellular signaling. This and other facets of the Hla-ADAM10 interaction remain to be explored through perturbation of ADAM10 in both the cellular and tissue context.

In the context of epithelial cells (such as pneumocytes and keratinocytes) that are key targets of α-toxin, E-cadherin is a principal substrate for ADAM10 [133,155]. Cleavage of E-cadherin by ADAM10 results in a loss of the homotypic interaction of the cadherin molecules on adjacent cells at the adherens junction, thereby injuring the epithelial tissue barrier function. *In vitro* studies demonstrated the surprising finding that treatment of epithelial cells with sublytic concentrations of α-toxin leads to a rapid upregulation of the metalloprotease activity of ADAM10, which in turn dismantled the adherens junction through cleavage of E-cadherin [11,12]. Extension of these studies in the context of infection revealed that α-toxin caused a primary disturbance of the epithelial barrier in the lung and the skin, manifesting as proteinaceous pulmonary edema and dermonecrotic injury, respectively. In both of these tissues, the α-toxin-ADAM10 complex was demonstrated to mediate E-cadherin cleavage *in vivo*, correlating with the physiologic evidence of epithelial injury observed during infection. These findings prompted examination of the molecular mechanism that underlies toxin-induced ADAM10 metalloprotease activation. Activation occurs at subcytolytic concentrations of α-toxin [11,12,13], however requires the formation of a fully assembled, patent heptameric pore on the surface of the cell. Three lines of evidence support this mechanism of activation: (1) the Hla_H35L_ mutant is unable to trigger ADAM10 activation—while this toxin variant displays normal ADAM10 binding [94], it is unable to form a stable oligomer on the cell surface and therefore is non-lytic [100]; (2) a “pre-pore locked” mutant harboring an engineered disulfide bond that tethers the stem domain to the globular cap, precluding insertion of the stem through the plasma membrane under oxidizing conditions, only causes ADAM10 activation in the presence of a reducing agent [11]; and (3) methyl-β-cyclodextrin, a small molecule that inserts into the open toxin pore, providing functional blockade of the pore, abrogates ADAM10 activation [11]. While the specific molecular mechanism of ADAM10 activation by α-toxin is not yet elucidated, the observed requirement for calcium in the extracellular media of toxin-treated cells is highly suggestive that the toxin pore functions as an ion channel permitting the influx of calcium into the cell [11]. It is known that ionophore treatment of cells provides a stimulus for activation of ADAM10 [140,156], likely through an “inside-out” signal relay. While the cytoplasmic domain of ADAM10 would seemingly be implicated in this signaling mechanism, deletion of this domain of ADAM10 does not fully impair ionophore-mediated ADAM10 activation [140], implying that other domains of the protein and possibly other signal transduction proteins play a cooperative role in ADAM10 activation.

Beyond the contribution of ADAM10-mediated cleavage of structural proteins that provide tensile strength to the tissue barrier, the α-toxin-ADAM10 interaction leads to the rapid dephosphorylation of FAK, paxillin, Src, and p130Cas, proteins integral to the establishment and maintenance of cellular focal adhesions that tether the cell to the basement membrane [94]. These cell signaling events are associated with dissolution of focal adhesions, defining a second molecular mechanism by which α-toxin contributes to tissue barrier disruption. Like ADAM10-mediated cadherin cleavage, focal adhesion disruption is observed at subcytolytic toxin concentrations, suggesting that an intracellular signal transduction cascade is potentially invoked. Together with data on the role of membrane lipids and ADAM10 in toxin binding, an integrated model can now be considered in which assembly of a multi-molecular protein receptor and signaling complex in a specific lipid microenvironment is required for optimal action of α-toxin.

Tissue barrier disruption is a hallmark of staphylococcal disease, manifest as injury to the skin, lung, mucous membranes, and vasculature (in the context of sepsis). Inoculation of α-toxin into the lungs of rabbits caused endothelial cell lysis and detachment from the basal membrane leading to endothelial barrier permeability and vascular leakage into the alveolar space [152]. In agreement with these observations, treatment of pulmonary endothelial cells with α-toxin induced the formation of large intercellular gaps associated with a decrease in barrier integrity [154]. Recent studies provide molecular insight on the nature of endothelial barrier disruption, demonstrating that α-toxin binding to ADAM10 on primary endothelial cells leads to rapid activation of the metalloprotease and cleavage of vascular endothelial (VE)-cadherin [13]. Toxin-deficient *S. aureus* strains display a virulence defect in a mouse model of lethal sepsis induced by intravenous inoculation of the pathogen; this correlates with toxin-mediated induction of endovascular injury and increased vascular permeability, as documented by dye extravasation studies [13].

**Figure 3:**
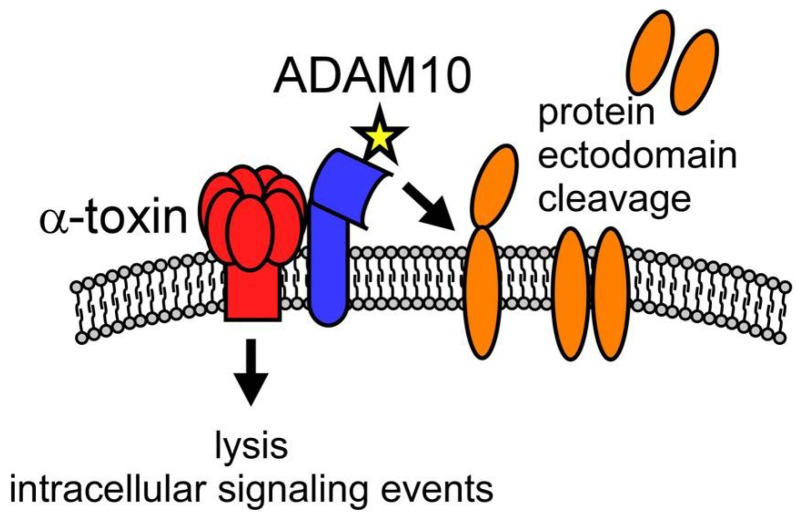
Dual mechanism of action of α-toxin on susceptible host cells. Model illustrating key functions of the α-toxin (red)-ADAM10 (blue) complex, facilitating membrane binding of the toxin with subsequent oligomerization and pore formation. The formation of the toxin pore leads to two functionally linked outcomes—induction of host cell signaling and/or cellular lysis (dependent on toxin concentration) and the rapid upregulation of the metalloprotease activity of ADAM10 (denoted by a star). ADAM10, in turn, acts in a cell-specific manner to cleave ectodomain-containing proteins (orange) that appear to represent important biological mediators of α-toxin action.

These observations hearken back to the early studies on the role of Hla in lethal disease and dermonecrotic injury, now providing molecular detail of α-toxin action in these states of host injury. It becomes clear that the functional outcome of the toxin-ADAM10 interaction is predicated both on the Hla-induced cell lysis and cell signaling, as well as on the tissue-specific actions of activated ADAM10 (Figure 3). The pleiotropic effects of a single toxin can, therefore, now be explained by three principles: (1) targeting of multiple host cell types through widespread expression of ADAM10; (2) cell-specific susceptibility to pore formation; and (3) diversity of cellular effects of the toxin dependent on the level of ADAM10 expression, the native functions of ADAM10 in the cell/tissue, and the intracellular signaling events elicited in the cell. Importantly, each of these outcomes are absolutely dependent on the assembly and patency of the α-toxin pore [11], demonstrating the primacy of the β-PFT structure for the full range of the toxin’s biologic activity. 

### 4.2. Toxin-Induced Immunomodulation

Multiple studies have indicated that immune cells are targets of α-toxin. While of interest, these studies have not yet led to a full appreciation for the role of the toxin in manipulating the immune response *in vivo* to facilitate pathogenesis. In recent years, several key studies have shed light on this topic, illustrating that α-toxin targets both innate and adaptive immune cells, altering the host response to staphylococcal infection and again demonstrating the diverse capabilities of the toxin. 

Pro-inflammatory signaling in the host in response to infection is a double-edged sword, affording protection from pathogens yet contributing to self-injury when overly robust. Inflammation is a key feature of *S. aureus* infection, most readily appreciated in the lungs and skin wherein the rapid infiltration of innate immune cells is observed in both human and murine hosts [157,158,159]. In murine pneumonia, α-toxin is required to generate a gradient of keratinocyte-derived chemokine (KC) and macrophage inflammatory protein-2 (MIP-2), CXC chemokines that facilitate neutrophil recruitment to the lung [160]. α-toxin induces inflammatory responses in multiple cells, resulting in the release of cytokines and vasoactive agents [21,148,149,150,151,161,162]. One hallmark of innate immune cell activation is the secretion of the inflammatory cytokine interleukin-1β (IL-1β), a consequence of inflammasome activation and caspase-mediated cleavage of pro-IL-1β to yield the active cytokine. Intoxication with α-toxin induces IL-1β secretion in macrophages and monocytes, implicating this lineage as a target of the toxin during infection and demonstrating the importance of inflammatory cell death in disease pathogenesis [9,31,148]. Craven and colleagues demonstrated that the nucleotide binding domain and leucine rich repeat containing gene family, pyrin domain containing protein (NLRP3) inflammasome was activated in monocytic cells following exposure to α-toxin, resulting in caspase-1 activation and IL-1β secretion [9]. While antagonism of the toxin *in vivo* through toxin-neutralizing antibodies blunted IL-1β secretion during *S. aureus* pneumonia [116], the molecular mechanisms underlying this response *in vivo* had not been investigated until recently. Following on the work of Craven, Kebaier and colleagues demonstrated toxin-dependent activation of the NLRP3 inflammasome in *S. aureus* pneumonia, leading to necrotic tissue injury [31]. Consistent with these findings, an attenuation of disease was observed in mice harboring germline deletion of *Nlrp3.* Mice lacking expression of NLRP3 display increased survival following intratracheal instillation of purified α-toxin, and show decreased lung pathology, IL-1β secretion and neutrophilic infiltrates upon infection with live staphylococci. These studies strongly implicate the role of NLRP3 and this inflammatory cascade as downstream effectors of α-toxin-mediated pathogenesis, linking this pathway to toxin-induced cell death that is most consistent with pyroptosis [9,163]. Interestingly, while antagonism of α-toxin by active vaccination, passive immunization, and direct small molecule inhibitors decreases the bacterial load in the lung during infection [107,116,164], mice harboring a deletion of either *Nlrp3* or conditional deletion of *Adam10* in the alveolar epithelium do not display a decreased bacterial burden following infection [11,31]. 

Illustrating the complexity of the host-pathogen interaction in distinct tissues, the induction of an IL-1β response to *S. aureus* skin infection is required for host immunoprotection, as mice lacking the ability to generate this inflammatory response through genetic deletion of the cytokine or its cellular receptor suffer exacerbated skin lesions in response to inoculation with *S. aureus* [10,165]. The establishment of an immunoprotective IL-1β response in the skin depends on neutrophil recruitment to the infection site, and elaboration of this cytokine by the neutrophil population [10]. α-toxin neutralizing antibodies significantly decrease the amount of IL-1β secreted by isolated mouse neutrophils exposed to Hla *in vitro*, suggesting that the toxin in part contributes to this beneficial inflammatory host response in the skin [10]. Taken together, these data illustrate the role of Hla in inflammasome activation, and highlights the dichotomy between the beneficial effect of this pro-inflammatory response to infection of the skin and the detrimental effect of toxin-mediated inflammation in the lung. Interestingly, antagonism of Hla by active or passive immunization affords protection in both the lung and the skin [30,107,108,116], highlighting the existing challenges in the field to understand how distinct cellular responses to α-toxin are integrated in the context of the tissue microenvironment during infection. 

In addition to these effects on innate immunity, there is growing evidence that α-toxin modulates the adaptive immune response. Patterning of adaptive immune responses have been noted to occur through two mechanisms: (1) direct cellular injury, wherein α-toxin induces apoptotic cell death in monocytes, B cells and T cells [22]; and (2) through alteration of signaling between innate and adaptive immune cells, particularly via the cytokine interleukin 17A (IL-17A). Treatment of human monocytes with subcytolytic concentrations of α-toxin stimulates secretion of IL-17A [166]. This cytokine polarizes the helper T cell response towards the induction of Th17 cells, a subset of CD4^+^ T cells that both respond to and express IL-17A, and contribute most notably to immunoprotection of the epithelium. The Th17 response has been implicated in both the immunopathogenesis of toxin-mediated inflammatory skin disease and in protection against acute infection [159,166,167]. Frank and colleagues recently used a whole-transcriptome approach to discern the effect of α-toxin on the host response to *S. aureus* pneumonia. This study compared responses in mice infected with wild-type *S. aureus* to those infected with toxin-deficient *S. aureus* in a murine disease model, demonstrating that toxin expression was associated with induction of the IL-17A response [29]. Mice infected with α-toxin-expressing *S. aureus* generate a T cell repertoire characterized by a greater number of IL-17A^+^ cells, illustrating a direct impact of the toxin on the adaptive immune system. While the precise molecular mechanisms that underlie induction of the polarized Th17 response are not yet known, the nucleotide-binding oligomerization domain containing 2 (NOD2) has been shown to play an important role in mediating innate immunodefense against *S. aureus*, as NOD2^−^^/−^ mice exhibit increased susceptibility to both intraperitoneal and subcutaneous *S. aureus* infection [127,168]. NOD2 functions as an intracellular receptor for peptidoglycan (PGN), thus the α-toxin pore is thought to enhance cytosolic access for this NOD2 ligand [127]. As NOD2 signaling can promote Th17 differentiation [169], this represents a plausible pathway for further investigation. 

While many of these observations suggest that the toxin’s harmful actions are unopposed by the host, epithelial cells are able to repair toxin-induced membrane injury and eliminate the toxin through a linked process of endocytosis from the membrane and exocytosis of the toxin in so-called “toxosomes”, or exosome-like vesicles [170,171,172]. Further, type-I interferon (IFN) produced by the host can afford protection against toxin-induced injury [126]. This effect is dependent on the presence of phospholipid scramblase I (PLSCR1), which provides protection against cellular leakage of ATP [128]. While the precise mechanisms of action of PLSCR1 are not yet elucidated, strong support for the role of this protein in host protection against α-toxin is observed in PLSCR1 knockout mice that demonstrate increased susceptibility to the injurious actions of the toxin in the lung [128]. The alteration of innate immune signaling pathways and the discovery of the ability of α-toxin to directly modulate the adaptive response is thus an exciting area of current research.

## 5. Conclusions and Future Directions

While *S. aureus* α-toxin has been among the most-studied bacterial cytotoxins, consideration of the knowledge gained over nearly a century of research highlights the extraordinary complexity of toxin function and illustrates many avenues for future investigation. The discovery of ADAM10 as a cellular receptor for α-toxin provides a number of opportunities to probe the biology of the toxin, enabling a focused examination of the effects of the toxin on specific cell populations in the context of disease. The broad expression pattern of ADAM10 raises several interesting areas for ongoing study that are necessary to define the principles that govern cell specificity of toxin action. We put forth several hypotheses in this regard: (1) the level of ADAM10 expressed on distinct primary cells may differ, forming the basis for relative susceptibility to α-toxin. This model is most consistent with existing data in the field noting a correlation between cell surface expression of ADAM10 and toxin-mediated lysis; (2) Cell specificity of α-toxin action is conferred by ADAM10 in concert with other proteins that display unique expression patterns, thereby providing some restriction to either toxin binding (such as a co-receptor) or susceptibility to lysis or ADAM10 activation (modulation of cell injury and signaling events); (3) Sensitivity to α-toxin may depend on ADAM10 expression as the cellular receptor in certain primary cell populations, while a distinct cellular receptor may exist on other cell populations. While the central role of ADAM10 expression has been confirmed in several epithelial tissues, primary endothelium, and red blood cells, further studies utilizing cell-type specific ADAM10 knockout approaches are anticipated to enable the most clear-cut investigation of this hypothesis. Importantly, this approach will not only examine the role of ADAM10 across distinct tissues, but will enable essential paired investigations on the role of the α-toxin-ADAM10 complex in disease pathogenesis. 

It is anticipated that further study of the toxin-ADAM10 complex will provide insight on the specific nature of the protein and lipid microenvironment that allows for toxin binding, assembly and host cellular signaling events generated during intoxication, enable investigation of human genetic polymorphisms that may increase susceptibility to disease, and facilitate studies of how Hla and other staphylococcal virulence factors act in concert to cause infection. As a number of studies in the field have highlighted features of cellular susceptibility to α-toxin *in vitro*, it will be essential to critically examine these observations *in vivo*, defining the molecular mechanisms of α-toxin-ADAM10 function and the role of other protein machinery in the context of *S. aureus* disease states. While of immediate relevance to our understanding of *S. aureus* disease, these studies will likely impact more broadly on our knowledge of pore-forming toxins and potentially highlight novel strategies to interfere with this family of toxins. 

Research over the last few years has led to a significant increase in our understanding of the role of α-toxin in the molecular pathogenesis of *S. aureus* disease. A tangible outcome of these studies is an appreciation of this toxin as a leading target for disease-modifying therapies, and has engendered an increased focus on understanding the role of the toxin in human *S. aureus* infection. Vaccines, passive immunization strategies, small molecule inhibitors of the toxin, and most recently small molecule-based targeting of host ADAM10 have all demonstrated a degree of efficacy in combatting *S. aureus* disease in animal modeling systems [11,12,13,30,105,108,116,119,121,164,173,174,175,176,177]. As such, many of these modalities are being developed for, and examined in, human clinical trials. The successful implementation of these preventatives and therapeutics will require an integrated understanding of the molecular pathogenesis of α-toxin-induced disease, the human clinical infection states in which Hla is essential, and an appreciation of the human immunologic responses that confer protection against toxin-mediated injury. Building on our wealth of knowledge about α-toxin, it now becomes a realistic expectation that modification of toxin-mediated *S. aureus* disease will be achieved in the coming years. 
